# 'I guess my own fancy screwed me over': transitions in drug use and the context of choice among young people entrenched in an open drug scene

**DOI:** 10.1186/1471-2458-10-126

**Published:** 2010-03-12

**Authors:** Danya Fast, Will Small, Andrea Krüsi, Evan Wood, Thomas Kerr

**Affiliations:** 1 British Columbia Centre for Excellence in HIV/AIDS, St Paul's Hospital, Vancouver, Canada; 2 Department of Medicine, Faculty of Medicine, University of British Columbia, Vancouver, Canada

## Abstract

**Background:**

There is growing interest in describing the broader *risk trajectories *experienced by young people who use drugs - that is, in describing the sequences of drug use transitions experienced by youth in relation to evolving understandings of risk and harm. This study sought to examine young people's perspectives regarding the evolution of their drug use in the context of a local drug scene in Vancouver, Canada.

**Methods:**

Semi-structured qualitative interviews with 38 individuals recruited from a cohort of young drug users known as the At-risk Youth Study (ARYS) were supplemented by ongoing ethnographic fieldwork (e.g., observations and informal conversations with youth) conducted within the same cohort population. Interviews were transcribed verbatim and a thematic analysis was conducted.

**Results:**

The majority of youth characterized past transition events as non-exceptional, largely 'spur-of-the-moment' decisions motivated by evolving feelings of curiosity. At the same time, participants' reflections indicated that the social, structural and material contexts of drug scene entrenchment play a powerful role in shaping these decisions and transition experiences.

**Conclusions:**

Importantly, as young people become increasingly entrenched in the local drug scene, drug use transitions seem to constitute increasingly relevant (and even 'inevitable') choices congruent with everyday lived experience. The implications of these findings for the development of meaningful interventions for youth are discussed.

## Background

Transitions into more harmful forms of illicit drug use among young drug users have been identified as an important focus for research and intervention [1,2]. In particular, given alarming rates of HIV and hepatitis C incidence among young drug users in several urban settings [3-5], a number of studies have focused on the transition into *injection *drug use among youth [6-9]. There is growing interest, however, in characterizing and understanding the broader *risk trajectories *experienced by young drug users, which include but are not limited to experiences with injection drug use [10-13]. A risk trajectories perspective emphasizes the sequences of transitions experienced by young people in relation to drug use and risk over time; furthermore, it recognizes that transitions are oftentimes shaped by particular critical moments (e.g., becoming homeless), as well as broader contexts (e.g., exclusion from mainstream opportunity structures) that can greatly influence long-term patterns of risk and harm.

While it is acknowledged that individual agency is intimately associated with drug-related risk taking [14], a growing body of research emphasizes the intersection of social, structural and physical environmental factors in powerfully shaping drug use practices among youth [9,11,15]. Attention to the *social situations*, *structures *and *places *in which risk is produced has illuminated some of the processes operating within drug-using contexts that *push *young people towards more harmful practices, until it becomes difficult or impossible for them to avoid 'risking risk' - that is, to avoid transitioning into increasingly harmful drug using behaviors [9,14,16]. From a *risk environment *perspective [17], social, structural and physical environmental factors are understood to be operating at three levels: the *micro- *or *interpersonal *level; the *meso-*level of social and group interactions (including institutional and organizational action or in-action); and the *macro*-level of core or distal causes that intersect with micro- and meso-level factors to produce risk. The risk environment is thus a product of *interplay *between different factors operating on multiple levels [18]. For example, intimate negotiations between drug users in particular places regarding specific drug use practices (micro-level factors) intersect with institutional responses - such as a lack of access to housing among the urban poor, and aggressive policing within public drug use settings that encourages the unsafe administration of drugs and equipment sharing among people who have *no where else to be but *in those settings - to shape patterns of risk and harm [19-21]. Social inequalities and wider cultural beliefs connected to racism, sexism, stigma and discrimination (macro-level factors) powerfully inform experience and practice at both the institutional level - where they are reproduced through policy (or the policy climate more generally) - and the interpersonal level - where they are oftentimes internalized and experienced as everyday features of lived experience in particular neighborhoods [22-24].

Increasing interest in risk environments has directed attention to the role played by *drug scenes *in shaping the evolution of risk and harm among specific drug using populations [25]. Broadly defined as distinctive inner-city areas characterized by high concentrations of drug users and drug dealing, drug scenes anchor elaborate social and spatial networks, practices associated with the day-to-day realities of securing basic necessities that go beyond drug procurement, and wider patterns of income generation activities. Drug scenes vary considerably according to a number of factors, including the types of drugs available, who controls the sale of illicit substances, the specific locales in which drugs are sold and used, as well as the history of particular drug-use settings [26,27]. 'Open' drug scenes are those in which drug procurement and use is highly visible and few barriers to access exist, while 'closed' drug scenes are those in which daily exchanges between various social actors are more clandestine, and individuals seeking drugs must know or be introduced to a dealer [28,29].

In Vancouver, Canada, the local street-based drug scene includes two distinctive neighborhoods known as the Downtown Eastside and the Downtown South. Although these areas are geographically adjacent (and within walking distance of each other), they are consistently differentiated according to their history and a number of aspects of place. Among the general public, the boundary that exists between them is largely aesthetic; while the Downtown Eastside is widely recognized as North America's poorest urban drug- and crime-ridden postal code [30,31], the Downtown South is a residential and entertainment district characterized by both high- and (limited) low-income housing and numerous thriving businesses. The drug-using populations in these two neighborhoods are also distinctive (although overlap exists); while the Downtown South is characterized by high rates of crystal methamphetamine sales and use primarily among youth [32], the Downtown Eastside is characterized by a trade in crack cocaine, cocaine and heroin involving primarily adult drug users, including many who inject drugs [33]. Furthermore, the Downtown Eastside is a long-standing and well-established drug market that has been in operation for decades, while the Downtown South drug market is a relatively recent development. Although the Downtown Eastside can accurately be characterized as a more 'open' drug scene in comparison to that of the Downtown South, in reality a wide range of illicit substances are easily available on the streets of both locales. Furthermore, both neighborhoods are characterized by thriving 'shadow economies' largely propelled by sex work activities, drug dealing and the exchange of stolen goods. The Downtown Eastside in particular has been subjected to intensive enforcement initiatives in recent years [34], although police activities are also ongoing in the Downtown South [35]. In spite of their differences, our ongoing ethnographic research in both neighborhoods indicates that a large number of young drug users move frequently *between *them.

In order to reflect an emerging focus on risk environments and the broader risk trajectories experienced by youth, this study sought to examine young people's understandings of how their drug use evolved over time in the context of the downtown Vancouver drug scene. We were particularly interested in how youth described their initiation into *self-identified *problematic drug use, as well as their subsequent progression towards what *they viewed *as increasingly harmful drug use practices. Thus, although the cessation of drug use is an important drug use transition in and of itself (and was featured in the narratives of a small number of participants), it does not form a part of the present discussion.

In the context of the present study, a 'transition' was defined as a *self-identified*, significant change in drug use practices (including initiation into drug use, and any subsequent changes in patterns of drug use including changes in substances used, modes of use, or intensity of use). From our previous ethnographic work in downtown Vancouver, we noted that young people often transition very quickly from one pattern of drug use practices to another and then back again (often over a period of just a few months). Thus, we were more interested in *how young people articulated *any meaningful transition in their drug use practices, than in whether these transitions led to 'regular' or 'robust' patterns of use according to etic definitions of the concept [1].

## Methods

We drew upon data from 38 in-depth individual interviews as well as ongoing ethnographic fieldwork (e.g., observations and informal conversations with youth) conducted in both the Downtown South and Downtown Eastside neighborhoods. Interviewees were recruited from the existing At-Risk Youth Study (ARYS) cohort, a prospective cohort of drug-using and street-involved youth that has been described in detail elsewhere [32]. Eligibility criteria for this larger cohort study includes being between the ages of 14 and 26 years and self-reported use of illicit drugs other than or in addition to marijuana in the past thirty days. Previous epidemiological research among this population indicates that they are vulnerable to intensive drug use - including the use of crystal methamphetamine, heroin, cocaine and crack cocaine [8,36,37] - homelessness [38], and alarming rates of HIV and hepatitis C infection [5].

A subgroup of the ARYS cohort was selected to complete qualitative interviews. A first wave of interviews was conducted during April and May of 2008, followed by a second wave of interviews in September and October of that year. Sampling was largely opportunistic; approximately half of the selected participants were well known to the research team as a result of our ethnographic activities, and an additional 20 participants were selected to ensure variation in gender, ethnicity, age, current housing and 'employment' situation, as well as length of time having lived within the downtown Vancouver drug scene comparable to that observed in the wider ARYS cohort. In addition to considerations of representativeness, the sample size was determined appropriate when it seemed that no dramatically new perspectives were emerging from the interviews or our ethnographic activities (although undoubtedly such views exist, and we do not purport to present a complete account of *all *youth perspectives regarding transitions in drug use in our setting). In other words, upon completing 38 interviews (supported by observational work and informal conversations with youth), we felt that a reasonably appropriate level of 'data saturation' had been achieved.

Interviews were undertaken by three trained interviewers (one male and two females) and facilitated through the use of a topic guide encouraging broad discussion of transitions in drug use within the downtown drug scene. More specifically, we asked youth to tell us about their first experiences with drug use which *in hindsight *they viewed as problematic, and then asked them how things progressed from there up until their present drug use practices (or lack thereof). We aimed to elicit broad narratives regarding the evolution of practices over time, when necessary probing for how specific drug use experiences were shaped by social, structural and physical environmental contexts (by asking for example, 'Who were you spending time with then?,' 'What was your living situation at that time?,' 'How were you getting money at that time?,' 'Can you describe the place where that happened?, ' etc.).

Interviews lasted between 30 and 120 minutes, were tape-recorded, transcribed verbatim and checked for accuracy. Shorter interviews (i.e. those less than one hour) were rare and usually due to the participant's ability (i.e., alertness, wakefulness) or willingness to participate in an interview lasting one hour or longer. Although such brief interactions cannot be considered 'in-depth interviews,' they oftentimes yielded important data and were therefore not removed from the data set. All participants provided informed consent, and the study was undertaken with ethical approval granted by the Providence Healthcare/University of British Columbia Research Ethics Board. Participants received a twenty-dollar honorarium. There were no refusals of the invitation to participate in the interview, and no dropouts (i.e. the participant chooses to decline participation in the study) occurred during the interview process.

Data collection and analyses occurred concurrently and via ongoing engagement with participants, in order to continually re-evaluate the validity of research findings. While remaining cognizant of confidentiality issues (many of the participants of this study knew each other and it was therefore important to emphasize that what one person said in an interview would under no circumstances be repeated to another participant), evolving interpretations of the data were discussed with participants, both informally with those who had already been interviewed, and more formally in subsequent interviews. This process was used to inform the focus and direction of subsequent interviews (for example, through the addition of new questions and probes). In addition, the research team discussed the content of the interviews throughout the data collection and analysis processes, informing the development and refinement of a coding scheme for partitioning the data categorically.

ATLAS.TI software was used to manage the coded data. Interview data was initially coded based on broad themes, including 'transition in substance used,' 'transition in intensity of use' and 'transition in mode of use.' In other words, we originally *separated out *different 'kinds' of transitions. However, from these broad categories emerged several more specific narrative themes that *cut across *the broader classifications. Furthermore, it was frequently the case that these 'types' of transitions occurred simultaneously in the lives of young people with whom we spoke and could not be meaningfully separated out, reinforcing the value of a broader approach to understanding drug use transitions among youth in our setting. We therefore revised our coding scheme to reflect the overarching narrative themes, which apply to a wide range of transitions experiences. All names appearing below have been changed, and some youth narratives have been condensed in the interest word length.

## Results

Participants ranged from 16 to 26 years of age and included 18 women, 18 men and 2 transgender individuals. Sixty-seven percent of study participants were Caucasian, 28 percent self-identified as being of Aboriginal descent, and 5 percent were African Canadian. Half of interview participants reported being homeless at the time of the study, and the majority had experienced homelessness at some time over the course of their involvement with the local scene. All but two participants were currently engaged in drug use practices that they defined as problematic (including the use of crystal methamphetamine, crack cocaine, cocaine and heroin), and over half of these participants had been involved in self-identified problematic drug use for at least three years. Participants were involved in numerous income generation activities (oftentimes simultaneously) including street-level drug dealing, sex work, theft and the exchange of stolen goods. To a lesser extent, some youth also engaged in recycling activities (referred to as 'binning'), panhandling, and street performing (referred to as 'busking'). In sum, the majority of the young people with whom we spoke were significantly 'entrenched' in the downtown drug scene; as characteristic of the wider ARYS cohort, they were largely consumed by the daily project of survival 'on the streets' in the context of homelessness, chronic poverty, involvement in harmful forms of drug use and/or dangerous income generation activities.

### Evolving curiosity and 'nonchalant' choices

Youth narratives regarding the evolution of their drug use emphasized several key themes. Perhaps most pervasive of these was the generalized assertion that transitions in drug use practices (including initiation into drug use) represent decision points over which individuals have *total *control. Young people articulated this sense of autonomy both when they were referring to their own transition experiences ('it was my choice totally - my friends had nothing to do with it') and when referring to the experiences of drug users in general ('they always have the choice - I hate it when people blame others for their mistakes'). Darren ran away from his foster home and became involved in the local drug scene at age 13 - before which time he described himself as being 'totally clueless' when it came to 'hard' drug use (unanimously defined by participants as the use of crystal methamphetamine, heroin, cocaine and/or crack cocaine). Upon arriving in Vancouver's Downtown Eastside neighborhood, Darren was homeless and immediately recruited as a street-level drug dealer - perhaps, he reflected, because of his age and relative immunity from police harassment. He attributed his initiation into crack cocaine use three weeks after arriving to growing curiosity about 'what the hell he was selling people.' Similarly, he described his transition from crack cocaine to crystal methamphetamine several *years *later as resulting from 'his own fancy' and a largely spur-of-the-moment decision:

*It was three years of using crack everyday until I just decided that I was going to do jib *[crystal methamphetamine] *one day. So it was my choice totally. Then I liked it, and that's the bad thing. That's always the bad thing - same as with the crack ... I guess my own fancy screwed me over big time in the end*. (Darren, age 23)

The majority of participants attributed transitions in their drug use practices to evolving curiosity and a resulting choice that *at the time *seemed relatively inconsequential; it was only in hindsight that these choices were sensationalized and recognized as having grave consequences.

A small number of youth, however, emphasized their limited autonomy in becoming involved with increasingly harmful forms of drug use, and the *inevitability *of progressing to 'the worst kind of drug use out there' (unanimously defined as intravenous heroin use). Marie described growing up 'on the streets' of the Downtown Eastside - a phrase that implies involvement with numerous outdoor *and *indoor locales with relevance to drug scene activities. During this time her parents were 'off doing heroin,' and she spent most of her time in the Downtown Eastside with the other *Lost Boys *(from the popular film title, i.e. other youth who grew up on the streets with parents who were heavily involved in the local scene). She reflected that she had 'been around drug use her whole life' whether via peers or her parents, and was 13 years old when she started smoking crystal methamphetamine in the Downtown South. Initially, she recalled avoiding injection drug use because she had 'seen what it had done to her parents.' Nevertheless, Marie described the circumstances that surrounded her transition to intravenous heroin use at age 15 - again emphasizing the role played by a growing sense of curiosity. However - in contrast to Darren's story - she described being fully aware of the long-term impact of her decision at the time; furthermore, she recalled a sense of resignation to the fact that 'kids like her' (i.e. youth who grew up on the streets) inevitably ended up as 'heroin addicts' and 'junkies':

*I finally got curious ... My friends were doing it and I was like, 'Well, my mom used to be a heroin addict too, so I want to try it' ... I thought, I don't know, I was probably always going to do it, because, like, I guess I was born addicted to it because of my parents and stuff.' *(Marie, age 16)

All participants made a direct connection between evolving curiosity in relation to a particular drug use practice and having repeatedly *watched *other social actors engaging in that practice. Even among young people who did not grow up on the streets, prolonged proximity to open drug use (which is particularly ubiquitous in Vancouver's Downtown Eastside neighborhood) was associated with the redefinition of previously established *risk boundaries *[14] and eventual engagement in forms of drug use that were previously viewed negatively. Carla started smoking crystal methamphetamine intensively with her boyfriend at age 16. Upon relocating to the Downtown Eastside at age 20 in order to 'find a cheap place to live,' she and her boyfriend transitioned to crack cocaine use because it was 'easy to get and it was everywhere.' Although heroin use is also ubiquitous in the Downtown Eastside, it remained 'off limits' for the pair during their first year downtown; as Carla recalled, she had always associated it with shameful and public 'junkie' behavior (as she put it, 'I didn't want to become one of those people in the park, nodding off with a needle hanging out of my arm'). However, when the couple lost their single-room occupancy hotel in the Downtown Eastside due to falling behind in rent, and her boyfriend ended up in jail, Carla described the growing sense of curiosity that led her to contemplate (and then go through with) snorting heroin for the first time:

*We always said we would NEVER do that *[heroin]*. Like never. But about four months ago, I don't know, I just started seeing people doing heroin and I just wanted to try it ... I mean, I had always been around it *[in the Downtown Eastside] *but I guess I just started to notice it more. I tried it, and I got addicted*. (Carla, age 22)

Furthermore, youth indicated that a range of social actors - including peers, romantic partners, dealers and 'clients' vis-à-vis drug dealing activities - could play an *active *role in the definition and re-definition of acceptable risk and 'normal' patterns of drug use. Anka became involved in the downtown drug scene when she was 13, at which time she was already using ecstasy. Like Marie, Anka had grown up around hard drug use (although not in downtown Vancouver) and initially avoided it as a result of negative childhood experiences. She recalled the circumstances under which she and a friend transitioned from ecstasy to crystal methamphetamine only a few months after she relocated to the streets of the Downtown Eastside:

*It used to be like me and my friend would get together, and we'd get some caps of E *[ecstasy]*... And then, after a while she was like 'Do you think maybe we could get jib *[crystal methamphetamine]*instead of E?' Just kind of nonchalant about it. At first I was like, 'Why do you want to get jib?', right? But then after a while I was kind of like, 'Oooh, yeah, I think that's a good idea.' *(Anka, age 19)

Although the transition from ecstasy to crystal methamphetamine could be interpreted as an *escalation *of stimulant use, it is important to note that participants rarely articulated these types of transitions as such. Rather, as noted above, the vast majority of participants stressed the (as Anka put it) 'nonchalant' circumstances under which they made the decision to transition from one substance to another. The exception was young people who 'grew up on the streets'; these youth tended to view their progression from marijuana to crack cocaine and/or crystal methamphetamine, and then finally to heroin, as an inevitable escalation towards 'the worst drug out there.' For the majority of participants, however, the notion of *escalating drug use *applied to transitions in mode of use - that is, transitions from smoking (understood as the least harmful) to snorting and then finally to injecting (understood as the most harmful). Youth consistently emphasized that these transitions in mode of use were almost always accompanied by transitions in *intensity *of use - and by association, an escalation of the harms associated with that particular substance.

### Contextualizing choice: the social-spatial and material circumstances of drug scene entrenchment

While curiosity and 'on-the-spot,' non-exceptional choices were associated with the *moment *of initiation or transition, participants' reflections in hindsight also pointed to the role played by social-spatial and material (i.e. economic) contexts in shaping these choices and prompting transitions to increasingly harmful forms of drug use. The role played by these wider contexts was underscored by those youth who had at one time attempted to exit downtown Vancouver and the local drug scene - oftentimes with the express purpose of transitioning *away *from harmful forms of drug use - and found that upon returning (whether by choice or material necessity or both), familiar social-spatial networks of drug users and dealers facilitated an immediate transition *back *into harmful drug use practices. For example, Anka lamented her rapid transition back into crystal methamphetamine use after a period of absence from downtown Vancouver when she was 16, indicating that upon her return she knew exactly *who to find *and *where to go *in order to get crystal meth:

*My mom sent me away to Armenia to live with my step-dad's family 'cause she wanted to get me off drugs ... When I got back from Armenia I just really needed to get high ... So I went over to this guy's house, because I knew that he always had jib *[crystal methamphetamine] *there*. *So that very first fucking day that I came back, I got really high on jib ... I have this little calendar that I kept up. I wrote down, like, the drugs that I did just cause I wanted to keep track ... the day that I came back *[from Armenia] *it says 'jib,' and then since that day, every single day it's full*. (Anka, age 19)

The relationship between the social-spatial and material contexts of drug scene entrenchment and transitions to increasingly harmful forms of drug use was perhaps most powerfully articulated by those participants who grew up *within *these contexts, and *without *the means to enact or even envision an exit from them (whether in the sense of exiting the physical, geographical boundaries of the drug scene, or the social structural circumstances embedded in these neighborhoods). Among these youth, initiation into drug use occurred at a very young age, and subsequent transitions to increasingly harmful forms of drug use were particularly accelerated. Both initiation and transition experiences were commonly facilitated by an older family member or long-time acquaintance who was themselves drug scene entrenched, and occurred in familiar places (e.g., concealed camps of people who are homeless in downtown Vancouver's Stanley Park, which is adjacent to the Downtown South). As noted previously, youth like Marie and Sara viewed their eventual transition to intravenous heroin use as a 'natural' - albeit deadly - progression:

*I was like eight. First time I ever smoked a joint*.

[DF: *And how did things progress from there?*]

*Well, smoked pot, drink a bit of alcohol at age 9-10, then I started getting into mushrooms, acid, you know, all the hallucinogens (and eventually with drugs as well). They were all around me down here, right? Then you get into the crystal methamphetamines by 12-13 *[years of age], *and then you get to the crack cocaine and then you get into the heroin, and then you're done. You're dead ... I started shooting *[injecting]*heroin when I was 15. We *[herself and the other *Lost Boys*] *were all at our camp in Stanley Park ... It just seemed like the thing to do - I mean, I grew up down here. And heroin's what I'm on right now*. (Sara, age 18)

Young people's immersion (or rapid re-immersion) in the social-spatial contexts of the local scene - combined with their *exclusion *from those places populated by upper and middle class citizens (contexts which might indeed offer a refuge from open drug use) - create a scenario in which youth increasingly viewed drug use as a relevant, mundane and even inevitable choice congruent with everyday lived experience. Within these social-spatial realities, drugs are both highly visible and highly available, while alternative contexts for escape and pleasure (e.g., involvement in recreational sport or arts programs) are completely out of sight - in both the literal and figurative sense.

Participants made a direct connection between drug use transitions and a material reality characterized by, on the one hand, chronic poverty and homelessness, and on the other, a heightened need to accrue income in order to remedy 'dopesickness' (i.e. withdrawal symptoms caused by escalating drug use). In our setting, these material conditions frequently intersect with the ubiquity of drug use and the availability of drugs (as well as the *absence *of opportunities to gain even low-level formal employment) to facilitate involvement in drug dealing activities, particularly when youth are relatively young and new to the scene. Drug dealing activities among youth in downtown Vancouver are most often informal and range from street-level dealing for relatively higher ranking 'workers,' to 'scoring' (i.e. buying) and re-selling small quantities of drugs for a modest profit. Importantly, these activities facilitate constant proximity to drugs and a *range *of drug use practices - including those not previously engaged in. Youth frequently hang out with or in the vicinity of their 'clients' (who are perhaps more accurately characterized as peers, friends, or casual acquaintances) while the latter get high, and may use drugs with them (whether they are engaged in the same drug use practices or not). This is especially the case if drug dealing activities are taking place at larger scale, 'multi-purpose' drug using locales such as 'crack shacks' (private residences where one can go to obtain and use drugs) or particular outdoor or semi-outdoor locales. When Lucas ran away to downtown Vancouver at age 14 he had no prior drug use involvement. On his first night in town he was introduced to crystal methamphetamine by 'some guy' he 'met on the street,' who took him to a park in the Downtown South that is also a major center of activity among young crystal methamphetamine users. A few months later he was homeless and began scoring and re-selling heroin to support his crystal methamphetamine use - an income generation strategy that eventually led to a transition in drug use practices:

*I was buying someone else's heroin and eventually I said, 'For once, you're going to let me try doing what you're doing, so at least I know what I'm buying ya!' ... Before that my friend would use heroin and I would use meth at this apartment where we would go. She didn't want me to try heroin, partly because, like, I didn't know much about drugs then. But I have a very strong curiosity in me, right? ... So I think that she saw at that moment I was going to try it no matter what*. (Lucas, age 25)

Alternatively, Shawna moved to the Downtown Eastside when she was 15, and soon after began selling drugs via her older sister's pre-existing connections to a well-established network of higher level dealers. These connections also provider her with a place to stay initially - she spent her first year downtown sleeping in the apartments of different men for whom she sold drugs (some of whom she became romantically involved with). She had no prior experience with drug use; however, once involved in drug dealing it was not long before she began 'doing her own product' (a scenario also described by Darren):

*I started selling drugs and when I'd finish, I'd go drinking in the bar *... [People in the bar started]*offering me lines of cocaine, so I started with that, and then I opened what I was selling - the crack. And, you know, I said, 'How do you do it?' and some chick showed me how, and I never went back*. (Shawna, age 19)

Shawna's experience - in which she was selling drugs for older men who were also providing her with a place to stay (and eventually with drugs as well) - points to yet another context that can greatly influence transitions in drug use, particularly among young women. In general, youth frequently reflected that in the context of the local scene, the distinction between a 'friend' and a 'drug dealer' is often unclear, and that these 'friend-dealers' could play a critical role in the transition from one kind of drug use to another. For example, Darren's 'spur of the moment' initiation into crack cocaine was largely facilitated by this type of social actor:

*Three weeks after I had gotten into town a friend of mine, well, my dealer, he turned around and went, 'Try this!' Hands me a pebble bowl of rock *[crack cocaine]*and, okay, I stuck it in the pipe, started smoking it and, oh my god. It was a dream come true ... I started a grand-a-day habit*. (Darren, age 23)

However, it seems that 'boyfriend-dealer' relationships are particularly prevalent in our setting for several reasons. Firstly, the material conditions experienced by young people in general can have especially adverse and oftentimes violent consequences for women - again, particularly when they are relatively young and new to the scene. In the context of unstable or non-existent housing (as was the case for Shawna), it is both protective and economically advantageous for young women to align themselves with men who are 'well connected' on the streets - and this almost always translates as well connected to drug dealing and drug procurement networks. Secondly, these boyfriend-dealer relationships are facilitated by the destabilized social networks frequently experienced by young women as a result of everyday incidents of arrest among young men entrenched in the local scene. The frequent incarceration of young men means that the young women who were formerly romantically attached to them must often seek out alternative social relationships during periods of the latter's incarceration, whether in order to secure greater safety, companionship and/or material resources (including drugs). These shifts in social networks - in which young women realign themselves with a new male partner who is also involved in drug dealing - frequently result in a corresponding shift in drug use patterns. As mentioned previously, Carla transitioned to heroin use shortly after her boyfriend was incarcerated. However, she transitioned to more intensive, injection heroin use after 'hooking up' with a new partner who was also a dealer:

*I met this other guy while my boyfriend was in jail ... he was a drug dealer and he gave me heroin every day, up to like four times a day ... That was when I really got into it. I started doing it more than ever and now I am wired to it *[physically dependent on it]. (Carla, age 22)

## Discussion and conclusions

Young people emphasized their autonomy in *choosing *to transition into particular drug use practices, whether out of curiosity or as a result of 'their own fancy.' Furthermore, they described the relatively *non-exceptional *circumstances of these choices at the moment when they were being made ('I just decided I was going to do it,' 'I just wanted to try it'). It was only in hindsight that young people *sensationalized *these moments as critical decision points ('I wish I had know then what I was getting into'), which greatly influenced longer-term patterns of risk and harm.

Undoubtedly, choice narratives were to a certain extent influenced by public health campaigns that assign responsibility for risky behavior on the individual actor and espouse an abstinence-based approach to avoiding transitions to more harmful forms of drug use (certainly, the youth with whom we spoke represent a highly researched and 'socially worked' population and are adept reiterating these public health messages in such a way that is pleasing to health workers and researchers). However, they also - somewhat paradoxically - reflect both assertions of *control *('it was *my *choice totally'), as well as young people's increasing *powerlessness *in the context of broader social structural forces related to poverty and social inequality. Farmer, Connors and Simmons [39] define *structural violence *as 'large scale forces - ranging from gender inequality to racism to poverty - which structure unequal access to good and services,' resulting in the social, emotional and physical conditions that produce risk. While not discounting young people's agency in shaping their drug use and the nature of their drug scene involvement, it is also recognized that forces of marginalization, stigmatization and other forms of structural violence can be internalized as everyday features of lived experience, and expressed at the individual level in the form of self-blame ('I guess my own fancy screwed me over big time') and fatalism ('I think that I was probably always going to do it') [11,18]. In this way, the structural violence of poverty and social inequality is produced and reproduced [39], while the ways in which power relations within societies hierarchies shape 'choice' and risk are obscured (including from young people themselves).

Furthermore, although youth emphasized a connection between evolving curiosity as a result of observing drug use behaviors and drug use transitions, our data contradicts the notion that harmful drug use among street-entrenched youth is attributable to a simple causal relationship between observation and imitation [40]. If this were the case, then any youth who walked though the Downtown Eastside or saw drug use in a graphic film like *Pulp Fiction *would be vulnerable. Rather, participant narratives illustrated the role played by intersecting social, spatial and material contexts in shaping the very experience of 'choice' over time. In the context of chronic poverty and ongoing social-spatial exclusion, young people come to 'notice' drug use differently (as Carla aptly described it). Not only are drugs highly visible within the physical landscape of the local drug scene, but drug scene entrenchment also provides the social-spatial and material contexts in which transitioning into drug use and into increasingly harmful practices becomes, over time, an obvious, 'nonchalant' or even inevitable choice, *particularly *in the context of exclusion from alternatives to this choice (e.g., exiting the local drug scene), which remain difficult to enact or sometimes *even envision*.

Consistent with previous work [12], our findings illustrate that within 'high risk' environments such as the drug scene described herein, risk assessments and drug-related decision making focus not on whether or not to take drugs, but rather on *acceptable *versus *unacceptable *forms of drug use - the definitions of which are continually being made and re-made through various social-spatial practices including interactions with other drug users, dealers, 'clients' and lovers 'on the streets.' Furthermore, as involvement in the local scene intensifies, these risk assessments and decisions are increasingly made in the context of escalating drug use and a corresponding need to accrue additional income via illicit income generation activities that 'push' youth towards more harmful drug use practices [41,42]. Each of these factors exacerbates the *social suffering *[43] experienced by young people, which likely further 'pushes' vulnerable youth towards harm. It is important to recognize that in the context of ongoing drug scene entrenchment, the risks inherent in transitions to increasingly harmful forms of drug use are only one part of the story, which *itself *needs to be contextualized within the myriad of risks inherent in experiences of chronic homelessness and poverty, marginalization and everyday violence among young people in our setting.

A risk environment perspective [17], points to several interconnected contextual factors (as well as gender dynamics) operating on different levels to powerfully shape transitions into increasingly harmful forms of drug use among local youth. At the micro-level, street-entrenchment and everyday interaction with the people and places that facilitate constant proximity to drug use and procurement activities, as well as the widespread availability of drugs, play a role in the re-definition of previously established 'risk boundaries' [14] around particular practices, and in the normalization of the most harmful forms of drug use [9]. Particularly among young women, unstable or non-existent housing, as well as the destabilization of social networks via aggressive police action that targets their male partners, can also each play a role in prompting transitions in drug use, as young women often find themselves quickly involved in new romantic relationships that include new patterns of drug use [11]. At the same time, macro-level factors such as entrenched poverty and social exclusion from mainstream opportunity structures encourage involvement in drug dealing and/or 'scoring' activities as one of the only means to daily survival within this setting, facilitating exposure to a range of drug use practices and transitions to more harmful forms of drug use. Furthermore, our results also indicate that childhood and early adolescent exposure to addiction (which is shaped by macro-level forces of entrenched poverty and social suffering in particular households and neighborhoods) often results in an accelerated risk trajectory, evidenced by transitions into the most harmful forms of drug use at an extremely early age. At the meso-level, the lack of social housing *combined *with drug treatment facilities - as well as a lack of recreation and education programs that might facilitate the construction of alternate subjectivities and new identities among youth *apart from *'drug user,' 'homeless,' or 'junkie' - mean that young people remain entrenched in the local scene without a viable exit strategy, and therefore highly vulnerable to transitioning into increasingly harmful forms of drug use.

Our findings illustrate that, in order to be effective interventions must provide accessible and attractive alternatives to what the street has to offer. It is only through access to *alternative *contexts and opportunities that young people entrenched in drug use settings will begin to re-envision and re-imagine the choices available to them. Furthermore, consistent with the findings of previous studies [27,44], our results indicate that interventions should aim to enable 'exit strategies' during the early stages of drug scene involvement, before the daily priorities associated with a lifestyle of addiction and survival make it *increasingly likely *that young people will transition into harmful forms of drug use, and *increasingly difficult or impossible *for them to envision or enact exits from this environment. In the short term, enabling environments [45] could include programs situated *outside of *the geographical boundaries of the local drug scene (such as recreation activities accompanied by accessible transportation to and from young people's places of primary residence), while in the long term, providing enhanced support in finding safe housing, income support and *meaningful *education and work training placements for young people is crucial. A more nuanced identification of the factors and wider environments that deter transitions into more harmful forms of drug use and/or enable youth to *exit *the drug scene during the early stages of street involvement is a crucial area for future research [45].

This study has several limitations that warrant acknowledgement. Our findings are based upon interviews with local youth participating in the current study. While an effort was made to ensure that the study sample reflects the demographics of the local youth drug using population, it became clear over the course of the research process that our sample is more representative of the *highest *risk youth in downtown Vancouver. It is notable that even our youngest participants (age 16) had relatively extensive experience with drug use at the time of interview. Further research is needed to examine the spectrum of risk experienced by local youth, and to understand why some young people abstain from harmful forms of drug use despite prolonged involvement in the local drug scene, while others feel virtually powerless to avoid transitioning into increasingly harmful practices. Finally, perhaps because of the social and economic marginalization experienced by *all *of our participants, potentially salient factors such as ethnicity and sexuality did not emerge as significant in our findings, although previous research within this population has demonstrated that these characteristics can intersect with other contextual factors to produce unique patterns of risk and harm [46-48].

In sum, youth described evolving curiosity and the everyday circumstances under which they chose to initiate drug use or transition into increasingly harmful drug use practices. However, for the participants of the present study, these 'everyday circumstances' included early and ongoing experiences of economic marginalization and social exclusion, unstable or non-existent housing, involvement in illicit income generation activities (most notably drug dealing), and immersion in shifting and often highly unstable social networks 'on the streets.' Importantly, they *excluded *access to the more mainstream opportunities for work, rest and recreation from a very early age. Our finding stress the need for a range of interventions that, on the one hand, enable youth in *navigating *drug-related harms within the local scene (including avoiding transitions to increasingly harmful forms of drug use), and on the other hand, enable them in *exiting *this setting altogether.

## Competing interests

The authors declare that they have no competing interests.

## Authors' contributions

DF, TK and WS designed the study. DF, TK and WS conducted the analysis of the data. DF prepared the first draft of the article. All authors contributed to the revision of the manuscript. All authors read and approved the final manuscript.

## Pre-publication history

The pre-publication history for this paper can be accessed here:

http://www.biomedcentral.com/1471-2458/10/126/prepub

## References

[B1] Strang J The study of transitions in the route of drug use: the route from one route to another Addiction 1992 87 3 473 483 10.1111/j.1360-0443.1992.tb01948.x 1559046

[B2] Vlahov D Updating the infection risk reduction hierarchy: Preventing transition into injection Journal of Urban Health 2004 81 1 14 19 15047779 10.1093/jurban/jth083 PMC3456135

[B3] Roy É HIV incidence among street youth in Montreal, Canada AIDS 2003 17 7 1071 1075 10.1097/00002030-200305020-00017 12700458

[B4] Doherty MC Correlates of HIV infection among young adult short-term injection drug users AIDS 2000 14 6 717 26 10.1097/00002030-200004140-00011 10807195

[B5] Miller CL HIV and Hepatitis C Outbreaks Among High-risk Youth in Vancouver Demands a Public Health Response Canadian Journal of Public Health 2005 96 2 107 108 10.1007/BF03403671 PMC6976065 15850028

[B6] Roy É Nonn E Haley N Transition to injection drug use among street youth-a qualitative analysis Drug and Alcohol Review 2008 94 19 29 10.1016/j.drugalcdep.2007.09.021 18077104

[B7] Roy É Drug injection among street youths in montreal: Predictors of initiation Journal of Urban Health 2003 80 1 92 105 12612099 10.1093/jurban/jtg092 PMC3456111

[B8] Wood E Circumstances of first crystal methamphetamine use and initiation of injection drug use among high-risk youth Drug Alcohol Rev 2008 27 3 270 6 10.1080/09595230801914750 18368608 PMC10464820

[B9] Harocopos A New injectors and the social context of injection initiation International Journal of Drug Policy 2009 20 4 317 323 10.1016/j.drugpo.2008.06.003 18790623 PMC2706152

[B10] Hser Y-I Longshore D Anglin MD The Life Course Perspective on Drug Use: A Conceptual Framework for Understanding Drug Use Trajectories Eval Rev 2007 31 6 515 547 10.1177/0193841X07307316 17986706

[B11] Bourgois P Prince B Moss A The Everyday Violence of Hepatitis C Among Young Women Who Inject Drugs in San Francisco Hum Organ 2004 63 3 253 264 16685288 10.17730/humo.63.3.h1phxbhrb7m4mlv0 PMC1458969

[B12] Mayock P Drug Pathways, Transitions and Decisions: The Experiences of Young People in an Inner-City Dublin Community Contemporary drug problems 2002 29 117 156

[B13] Elder GH Elder GH Perspectives on the life course Life course dynamics 1985 Cornell University Press: Ithaca, New York 23 49

[B14] Mayock P Scripting risk: Young people and the construction of drug journeys 2005 12 5 Drugs: education, prevention and policy 349 368

[B15] Small W Public injection settings in Vancouver: Physical environment, social context and risk International J Drug Policy 2007 18 27 36 10.1016/j.drugpo.2006.11.019 17689341

[B16] Lovell AM Risking risk: the influence of types of capital and social networks on the injection practices of drug users Social Science & Medicine 2002 55 5 803 821 10.1016/s0277-9536(01)00204-0 12190272

[B17] Rhodes T The 'risk environment': a framework for understanding and reducing drug-related harm International Journal of Drug Policy 2002 13 2 85 94 10.1016/S0955-3959(02)00007-5

[B18] Rhodes T The social structural production of HIV risk among injecting drug users Social Science & Medicine 2005 61 1026 1044 10.1016/j.socscimed.2004.12.024 15955404

[B19] Maher L Dixon D Policing and public health: Law enforcement and harm minimization in a street-level drug market Brit J Criminol 1999 39 4 488 512 10.1093/bjc/39.4.488

[B20] Shannon K Mapping violence and policing as an environmental-structural barrier to health service and syringe availability among substance-using women in street-level sex work International Journal of Drug Policy 2008 19 2 140 147 10.1016/j.drugpo.2007.11.024 18207725

[B21] Kerr T Small W Wood E The public health and social impacts of drug market enforcement: A review of the evidence International Journal of Drug Policy 2005 16 4 210 220 10.1016/j.drugpo.2005.04.005

[B22] Fast D Safety and danger in downtown Vancouver: Understandings of place among young people entrenched in an urban drug scene Health & Place 2009 16 1 51 60 10.1016/j.healthplace.2009.07.004 PMC2783406 19733496

[B23] Bourgois P Schonberg J Intimate apartheid: Ethnic dimensions of habitus among homeless heroin injectors Ethnography 2007 8 1 7 31 10.1177/1466138107076109 19777125 PMC2748417

[B24] Bourdieu P Wacquant L An invitation to reflexive sociology 1992 Cambridge: Polity

[B25] Hough M Natarajan M Hough M, Natarajan M Introduction: Illegal drug markets, research and policy Illegal drug markets: from research to policy 2000 Criminal Justice Press: Monsey, NJ 1 18

[B26] Bourgois P In Search of Respect: Selling Crack in El Barrio 1996 Cambridge: Cambridge University Press

[B27] Maher L Sexed work: Gender, race and resistance in a Brooklyn drug market 1997 New York: Oxford University Press

[B28] May A Serving Up: The impact of low-level police enforcement on drug markets 2000 Home Office: London

[B29] May A Hough M Illegal dealings: The impact of low-level police enforcement on drug markets European Journal on Criminal Policy & Research 2001 9 2 137 162

[B30] Strathdee SA Needle exchange is not enough: lessons from the Vancouver injecting drug use study Aids 1997 11 8 F59 65 10.1097/00002030-199708000-00001 9223727

[B31] Wood E Factors associated with persistent high-risk syringe sharing in the presence of an established needle exchange programme Aids 2002 16 6 941 3 10.1097/00002030-200204120-00021 11919503

[B32] Wood E Evaluating methamphetamine use and risks of injection initiation among street youth: the ARYS study Harm Reduct J 2006 3 18 10.1186/1477-7517-3-18 16723029 PMC1481558

[B33] Wood E Kerr T What do you do when you hit rock bottom? Responding to drugs in the City of Vancouver International Journal of Drug Policy 2006 17 2 55 60 10.1016/j.drugpo.2005.12.007

[B34] Small W Impacts of intensified police activity on injection drug users: Evidence from an ethnographic investigation International Journal of Drug Policy 2006 17 85 95 10.1016/j.drugpo.2005.12.005

[B35] The McCreary Centre Society Between the cracks: Homeless youth in Vancouver 2002 The McCreary Centre Society: Vancouver

[B36] Lloyd-Smith E High prevalence of syringe sharing among street involved youth Addiction Research & Theory 2008 16 4 353 358

[B37] Werb D Nonfatal Overdose Among a Cohort of Street-Involved Youth Journal of Adolescent Health 2008 42 3 303 306 10.1016/j.jadohealth.2007.09.021 18295139 PMC2290001

[B38] Rachlis BS High rates of homelessness among a cohort of street-involved youth Health & Place 2008 15 1 10 17 10.1016/j.healthplace.2008.01.008 PMC2606292 18358759

[B39] Farmer P Connors M Simmons J Women, poverty and AIDS: sex, drugs and structural violence 1996 Monroe, Maine: Common Courage Press

[B40] Moore D Opening up the cul-de-sac of youth drug studies: a contribution to the construction of some alternative truths Contemporary Drug Problems 2002 29 1 13 63

[B41] Werb D Risks Surrounding Drug Trade Involvement Among Street-Involved Youth The American Journal of Drug and Alcohol Abuse 2008 34 6 810 820 10.1080/00952990802491589 19016187

[B42] Kerr T Characteristics of injection drug users who participate in drug dealing: implications for drug policy Journal of Psychoactive Drugs 2008 20 2 147 152 10.1080/02791072.2008.10400624 18720663

[B43] Kleinman A Das V Lock M Social Suffering 1997 Berkeley: University of California Press

[B44] Bungay V Life with jib: A snapshot of street youth's use of crystal methamphetamine Addiction Research & Theory 2006 14 3 235 251

[B45] Moore D Dietze P Enabling environments and the reduction of drug-related harm: re-framing Australian policy and practice Drug and Alcohol Review 2005 24 3 275 284 10.1080/09595230500170258 16096131

[B46] Miller CL Females Experiencing Sexual and Drug Vulnerabilities Are at Elevated Risk for HIV Infection Among Youth Who Use Injection Drugs J Acquir Immune Defic Syndr 2002 30 335 341 12131571 10.1097/00126334-200207010-00010

[B47] Strathdee SA Social determinants predict needle-sharing behaviour among injection drug users in Vancouver, Canada Addiction 1997 92 10 1339 47 10.1111/j.1360-0443.1997.tb02852.x 9489050

[B48] Wood E Burden of HIV Infection Among Aboriginal Injection Drug Users in Vancouver, British Columbia Am J Public Health 2008 98 3 515 519 10.2105/AJPH.2007.114595 18235063 PMC2253561

